# Artificial Intelligence in Pharmacoepidemiology: A Systematic Review. Part 1—Overview of Knowledge Discovery Techniques in Artificial Intelligence

**DOI:** 10.3389/fphar.2020.01028

**Published:** 2020-07-16

**Authors:** Maurizio Sessa, Abdul Rauf Khan, David Liang, Morten Andersen, Murat Kulahci

**Affiliations:** ^1^ Department of Drug Design and Pharmacology, University of Copenhagen, Copenhagen, Denmark; ^2^ Department of Applied Mathematics and Computer Science, Technical University of Denmark, Lyngby, Denmark; ^3^ Department of Business Administration, Technology and Social Sciences, Luleå University of Technology, Luleå, Sweden

**Keywords:** systematic review, pharmacoepidemiology, artificial intelligence, machine learning, deep learning

## Abstract

**Aim:**

To perform a systematic review on the application of artificial intelligence (AI) based knowledge discovery techniques in pharmacoepidemiology.

**Study Eligibility Criteria:**

Clinical trials, meta-analyses, narrative/systematic review, and observational studies using (or mentioning articles using) artificial intelligence techniques were eligible. Articles without a full text available in the English language were excluded.

**Data Sources:**

Articles recorded from 1950/01/01 to 2019/05/06 in Ovid MEDLINE were screened.

**Participants:**

Studies including humans (real or simulated) exposed to a drug.

**Results:**

In total, 72 original articles and 5 reviews were identified *via* Ovid MEDLINE. Twenty different knowledge discovery methods were identified, mainly from the area of machine learning (66/72; 91.7%). Classification/regression (44/72; 61.1%), classification/regression + model optimization (13/72; 18.0%), and classification/regression + features selection (12/72; 16.7%) were the three most frequent tasks in reviewed literature that machine learning methods has been applied to solve. The top three used techniques were artificial neural networks, random forest, and support vector machines models.

**Conclusions:**

The use of knowledge discovery techniques of artificial intelligence techniques has increased exponentially over the years covering numerous sub-topics of pharmacoepidemiology.

**Systematic Review Registration:**

Systematic review registration number in PROSPERO: CRD42019136552.

## Introduction

**Figure 1:**
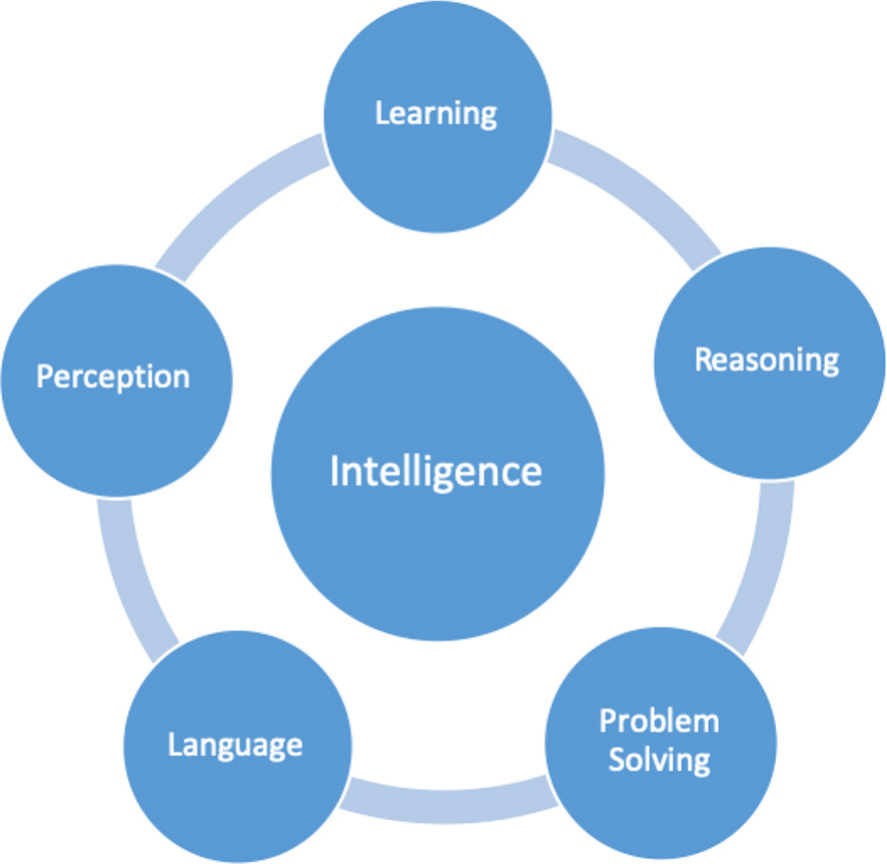
Artificial intelligence abilities.

By definition, artificial intelligence is “*the theory and development of computer systems able to perform tasks normally requiring human intelligence*” (Oxford, 2019). The British logician Alan Turing reports the earliest work in the field in the second quarter of the 20th century. In 1935, Alan Turing proposed the basic concept of an intelligent machine commonly known as universal Turing Machine. He further elaborated his vision in 1947 by describing computer intelligence as “*a machine that can learn from experience*” (Turing, 1937). As human intelligence is a combination of diverse abilities (i.e., learning, reasoning, problem solving, perception, and using language), artificial (or machine) intelligence is also a composite of methods and techniques from different disciplines of science and engineering to assimilate them in machines (**Figure 1**). It is worthy to note that artificial intelligence is commonly confused with machine learning. Learning (Machine/Deep Learning) is a subfield in artificial intelligence that deals with methods and techniques to assimilate learning abilities in machines. One reason of machine (or deep) learning emerging as a dominant sub-field of artificial intelligence is the considerable advancement in computer technologies and impressive achievements in learning algorithms. By definition, machine learning is a multidisciplinary field, which involves methods and techniques from mathematics, statistics, and computer science to learn from experiences (historical data) with respect to some tasks (i.e., the nature of the problem), and measure the performance (performance matrix) and improve it (re-enforcement) (Michie et al., 1994). Today, machine learning algorithms based on the principal of reinforcement learning not only enhances the learning abilities of the machine but also complement the other aspects of intelligence such as appropriate reasoning, efficient problem solving, and factual perception. Traditionally, experimental design, observational data analysis (statistical data analysis), and computer science have always been integral constituents of research in biomedical sciences. However, in the past decade the sprightly ascent of machine learning based knowledge discovery methods in artificial intelligence sparked this trend conspicuously. For numerous medical fields, the contribution of knowledge discovery techniques in artificial intelligence have been described extensively. However, their level of infusion to pharmacoepidemiology is unknown. Acording to the international society of pharmacoepidemiology, this discipline may be defined as “*the study of the utilization and effects of drugs in large numbers of people.*” Considering this gap in knowledge, the objective of this systematic review is to provide an overview of the use of knowledge discovery techniques of artificial intelligence in pharmacoepidemiology.

## Methods

An independent author (MS) registered the protocol of the systematic review in the PROSPERO International Prospective Register of Systematic Reviews database (identifier CRD42019136552).

### Eligibility Criteria for Considering Studies in This Review

We evaluated observational studies, meta-analyses, and clinical trials using artificial intelligence techniques and for which the exposure or the outcome of the study was a drug. Drugs include any substance approved on the pharmaceutical market having an anatomical therapeutic chemical classification code as proposed by the World Health Organization (WHO). Only studies for which the full text was available in the English language were considered as eligible. Abstracts sent to international or national conferences, letters to the editor, and case reports/series were considered ineligible along with articles evaluating natural language processing techniques. Reviews describing the use of natural language processing techniques are available elsewhere (Dreisbach et al., 2019). The reference list of narrative and systematic reviews included with our MEDLINE query were further screened for undetected records.

### Outcome

The main outcome was the frequency of studies published per year from January 1950 to May 2019, a narrative overview of their findings, and a lay description of knowledge discovery methods of artificial intelligence that were used. Secondary outcomes included the evaluation of 1) the medical field in which the aforementioned techniques were used and 2) the number and the type of artificial intelligence techniques that were used. Additionally, we assessed the frequency distribution of articles by 3) the study design; 4) type of data sources (e.g. primary/secondary or simulated); 5) the specific data source; 6) the purpose for using artificial intelligence based knowledge discovery techniques, and 7) the level of evidence provided by the study.

The purpose of using artificial intelligence based knowledge discovery techniques (outcome no. 6) was categorized as follows: 1) To predict clinical response following a pharmacological treatment; 2) To predict the needed dosage given the patient’s characteristics; 3) To predict the occurrence/severity of adverse drug reactions; 4) To predict diagnosis leading to a drug prescription; 5) To predict drug consumption, 6) To predict the propensity score; 7) To predict drug-induced lengths of stay in hospital; 8) To predict adherence to pharmacological treatments; 9) To optimize treatment regimen; 10) To identify subpopulation more at risk of drug inefficacy, and 11) To predict drug-drug interactions.

### Search Methods for the Identification of Studies

Ovid MEDLINE (from January 1950 to May 2019) was searched along with the references listed in the reviews identified with our research query (**Supplementary Table 1**). Preferred Reporting Items for Systematic Reviews and Meta-Analyses (PRISMA) checklist is provided in **Supplementary Table 2**.

### Selection of Studies

In the first screening procedure, titles and abstracts of retrieved record were screened by two independent researchers (MS and DL) for obvious exclusions. All articles that were considered eligible at the first screening procedure underwent a full-text evaluation. If disagreements arose during the two steps evaluation process, it was resolved by consensus.

### Data Extraction and Management

A data extraction form was developed for this systematic review and it is shown in **Supplementary Table 3**. The scale proposed by Merlin et al. (2009) was used to establish the level of evidence of each study.

## Results

**Figure 2:**
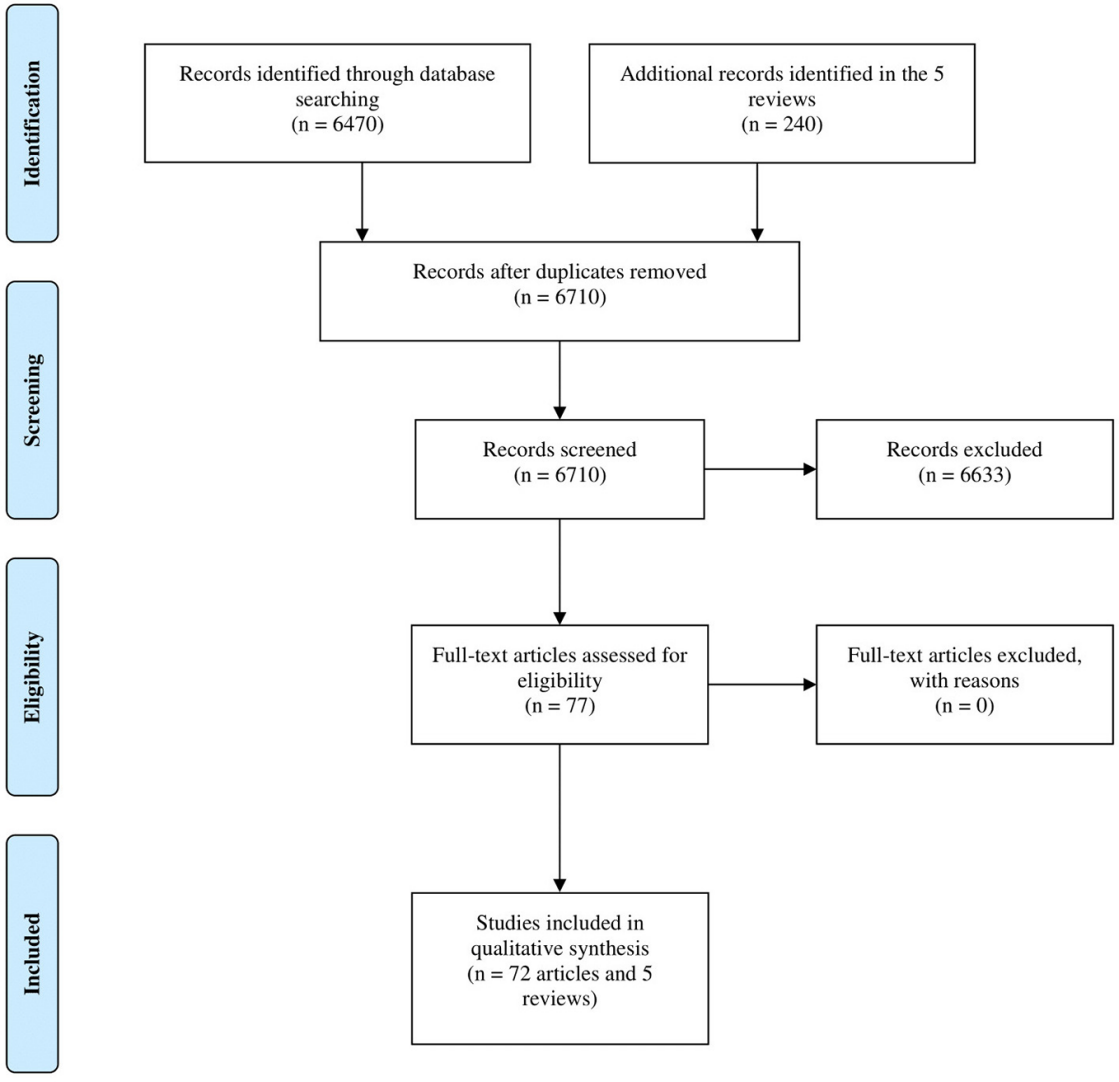
Study flow diagram.

In total, 6,470 and 240 records were identified in Ovid MEDLINE and in the reference list of reviews retrieved with the search query, respectively. After title/abstract screening, 6,633 records were eliminated because of ineligibility and 77 articles (72 original articles and 5 reviews) underwent a full-text evaluation. The 77 articles were considered eligible to be included in this systematic review. The PRISMA flowchart of the selection process is shown in **Figure 2** and the PRISMA checklist has been provided in **Supplementary Table 2**.

**Figure 3:**
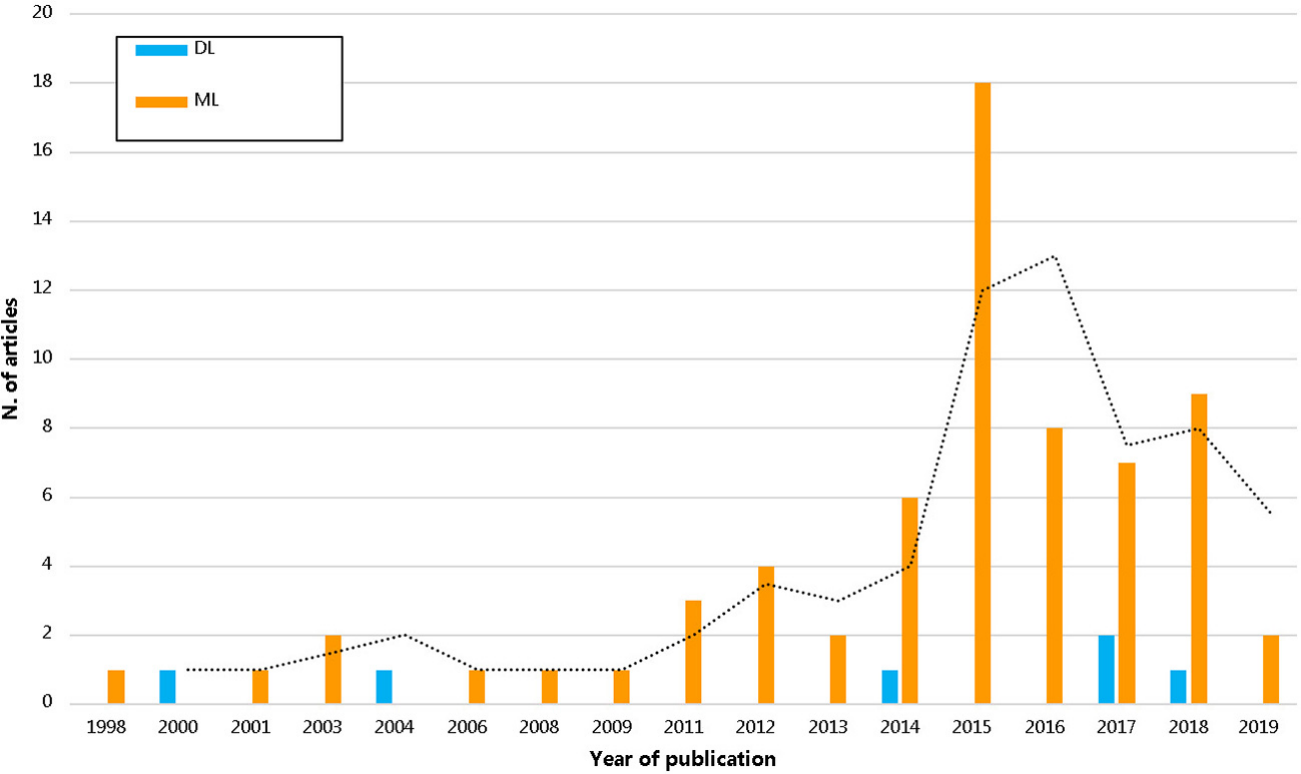
The trend of pharmacoepidemiological studies using artificial intelligence by years. DL, deep learning; ML, machine learning.

We observed increased use of artificial intelligence based knowledge discovery techniques in pharmacoepidemiology over the years as seen in **Figure 3**. In all, 17 medical fields were identified. The top four most prevalent medical fields were pure pharmacoepidemiology (16/72; 22.2%), oncology (15/72; 20.8%), infective medicine (8/72; 11.1%), and neurology (6/72; 8.3%) (**Supplementary Table 4**).

Fifty-five out of 72 articles (76.4%) used artificial intelligence techniques in the setting of a cohort study (**Supplementary Figure 1**). Most of the studies provided a medium-low level of evidence of III-3 (4/72; 5.6%), III-2 (49/72; 68.1%), and III-1 (16/72; 22.2%) while, a few articles provided a level of evidence of II (3/72; 4.1%).

In the 72 selected articles, the data sources included electronic health records (36.1%), *ad-hoc* databases from clinical studies (31.9%), administrative databases (29.2%), survey (1.4%), and simulated data (1.4%). The data sources were mainly secondary (59.8%) and primary sources (31.8%). Only in two articles (2.8%), researchers used both secondary sources and simulated data. Analogously, only in two articles (2.8%), researchers used simulated data (2.8%). The specific data sources used in selected articles are provided in **Supplementary Table 5**.

### Main Applications of Knowledge Discovery Techniques in Pharmacoepidemiology

A narrative overview of the articles is provided in **Table 1**. The lay description of the knowledge discovery techniques that were used in retrieved articles is provided in *Lay Description of the Knowledge Discovery Techniques of Artificial Intelligence Used in Pharmacoepidemiology*.

**Table 1:** Main applications of knowledge discovery methods of artificial intelligence (AI) in pharmacoepidemiology.

AI technique	Application	Study/ies	AI used for feature selection	AI used for model optimization
Artificial Neural Network	To predict the clinical response following a pharmacological treatment	1) Barbieri et al. used an artificial neural network to predict future hemoglobin levels among patients with end-stage renal disease that received pharmacological treatment for anemia (Barbieri et al., 2015) 2) The aforementioned statistical model was also used by Snow et al. to predict the presence or absence of cancer in patients that underwent laparotomy and chemotherapy for stages III or IV ovarian cancer. In particular, artificial neural networks provided a better prediction of the presence/absence of cancer than standard logistic/linear regression analyses (Snow et al., 2001). 3) Buchner et al. found that used an artificial neural network to predict metastatic renal cell carcinoma in patients with renal cell carcinoma (Buchner et al., 2012). 4) Saadah et al. have used artificial neural networks to identify the subpopulation of premature infants that benefitted of pharmacological prophylaxis for respiratory syncytial virus with palivizumab. In particular, the authors found that the statistical method was able to identify two main features i.e. extreme low-birth weight male infants and congenital heart disease as key elements for the effectiveness of the treatment (Saadah et al., 2014). 5) The Artificial neural network technique was used by Kebede et al. to predict the change in CD4 count among patients who underwent antiretroviral treatment. The model was found less effective than other machine learning techniques (Kebede et al., 2017). 6) Schmitz et al. used a neural network model to identify genetic markers for treatment success in heart failure patients (Schmitz et al., 2014). The model provided the fourth best accuracy when compared to other machine learning techniques used by the researchers. 7) Hardalaç et al. used a neural network model to evaluate the impact of azathioprine treatment on mucosal healing (Hardalac et al., 2015). 8) Albarakati and colleagues used an artificial neural network to classify genes as interacting or not interacting with BRCA-1DNA repair gene among patients underwent to the pharmacological treatment with cisplatin for breast cancer (Albarakati et al., 2015).	5) Yes	
	To predict the needed dosage given the patient’s characteristics	1) Urquidi-Macdonald and colleagues used a back-propagation neural network to individualize dosing for drugs with a narrow therapeutic index like abciximab to prevent adverse drug reactions. In particular, they combined information from abciximab dosage, patient sociodemographic characteristics, clinical history, and abciximab *ex vivo* platelet aggregation for predicting the dosage (Urquidi-Macdonald et al., 2004). 2) Tang et al. used an artificial neural network and other machine learning techniques to predict tacrolimus dose in patients undergoing renal transplantation (Tang et al., 2017). 3) Liu et al. used an artificial neural network in comparison with other machine learning techniques or multiple linear regression to predict the pharmacogenetic-guided dosage of warfarin (Liu et al., 2015). 4) Li and colleagues evaluated the efficiency of artificial neural network in comparison with multiple linear regression for the pharmacogenetic-guided dosage of warfarin discovering that for Chinese patients, the multiple linear regression gave the lowest mean absolute error (Li et al., 2015). 5) Saleh et al. found that an Elman artificial neural network was a reliable technique for predicting warfarin dosage in the clinical setting of dosage individualization (Saleh and Alzubiedi, 2014). 6) For African-American patients, the abovementioned statistical model was not able to improve the predictive performance of the dosing algorithm, except that for patients requiring a dose equal or greater than 49 milligrams per week (Alzubiedi and Saleh, 2016).		4) Yes
	To predict the occurrence/severity of adverse drug reactions.	1) Keijsers and colleagues found that the neural network was able to assess the severity of levodopa-induced dyskinesia in patients with Parkinson’s disease. The model performance was reliable considering that it misclassified in a few cases when compared to those assessed by the physicians (Keijsers et al., 2003). 2) Artificial neural networks were used to identify laboratory event-related adverse drug reactions in electronic health records. The model had the highest sensitivity and negative predictive value among several machine-learning techniques (e.g. random forest, support vector machine, regularized logistic regression, etc.) to predict the study outcome. 3) In the study conducted by Hoang et al, the authors assessed sequences of drug redemptions as proxies for adverse drug reactions. The artificial neural network performed inadequately for this classification task (Hoang et al., 2018). 4) Li et al. used the model to identify levodopa-induced dyskinesia in patients with Parkinson disease (Li et al., 2017). 5) Jeong et al. used an artificial neural network technique to predict adverse drug reactions in electronic healthcare records by using laboratory results as potential predictors (Jeong et al., 2018).	4) Yes	3) Yes 5) Yes
	To predict diagnosis leading to a drug prescription.	1) Artificial neural networks have been used by Rezaei-Darzi et al. to predict the labeling diagnosis leading to a pharmaceutical prescription. This statistical model was able to predict this diagnosis in 93.3% of cases showing very high accuracy (Rezaei-Darzi et al., 2014).		1) Yes
	To predict drugs consumption	1) Hu and colleagues found that artificial neural networks performed worse than decision tree-based learning in predicting drugs consumption for analgesia in a cohort of 1099 patients where more than 270 have been used to train the statistical model (Hu et al., 2012). 2) Smith et al. used a multilayer perceptron neural network to predict anticoagulation in patients in hemodialysis (Smith et al., 1998).		1) Yes 2) Yes
	To predict the propensity score	1) Setoguchi and colleagues found that this when compared to standard logistic regression, artificial neural network provide the least biased estimates of the propensity score in many clinical scenarios (Setoguchi et al., 2008).		
	To predict drug-induced lengths of stay in hospital	1) Kim and colleagues, instead, found analytic advantages of using artificial neural network instead of logistic regression for predicting lengths of stays in the post-anesthesia care unit following general anesthesia (Kim et al., 2000).		
Auto-contractive maps	To predict the clinical response following a pharmacological treatment	1) In the article from Podda et al., auto-contractive maps were used to predict platelet reactivity in clopidogrel-treated patients given a set of demographic and clinical information.		
Random forest	To predict the clinical response following a pharmacological treatment	1) LaRanger et al. found that the random forest was an efficient machine learning technique to identify genes that could predict response to keloid treatment with 5-fluorouracil (LaRanger et al., 2019). 2) Li et al. used a random forest model to predict that factors that increased the probability or the reduction of brain edema in patients treated with bevacizumab that underwent radiation therapy for nasopharyngeal carcinoma. The predictors selected by the random forest were able to provide a good predictive power (84% area under Receiving Operator Characteristic curve) (Li et al., 2018). 3) Devitt et al. used a random forest model to identify features in early proteomic spectra that predict the response to treatment with PEGylated interferon a-2b and ribavirin in patients with hepatitis C (Devitt et al., 2011). 4) Schmitz et al. used clinical and genetic variables to classify patients as responders/non-responders to cardiac resynchronization therapy. The random forest was one of the top four best models in terms of specificity, sensitivity, and accuracy for predicting the outcome (Schmitz et al., 2014). 5) Waljee et al. used a random forest to predict the clinical remission for patients with inflammatory bowel disease treated with thiopurines. Researchers used laboratory values and age as predictors. The model classified correctly patients in remission with an area under Receiving Operator Characteristic curve of 79% (95%CI 0.78-0.81) (Waljee et al., 2017). 6) Sangeda et al. used a random forest to predict the occurrence of virological failure in patients treated with antiretroviral drugs for HIV (Sangeda et al., 2014). 7) Kebede et al. used a random forest to predict CD4 count changes and to identify predictors of such change in patients with HIV/AIDS. When compared to other machine learning algorithms as J48 (accuracy 98.69%) or support vector machine (accuracy 96.62%), the random forest provided the best prediction model for CD4 count changes (accuracy 99.98%) (Kebede et al., 2017). 8) In the article from Podda et al, a random forest was used to predict platelet reactivity in clopidogrel-treated patients given a set of demographic and clinical information (Podda et al., 2017). 9) Albarakati et al. used a random forest model to predict genes that were expressed differently in patients with mRNA BRCA1+ and mRNA BRCA1− to assess their impact on prognosis (Albarakati et al., 2015). 10) Pusch et al. used a random forest model to identify predictors of all-cause mortality in patients with extra-pulmonary tuberculosis (Pusch et al., 2014).	3) Yes 7) Yes	
	To predict the needed dosage given the patient’s characteristics	1) Tang et al. used a random forest model and other machine learning techniques to predict tacrolimus dose in patients undergoing renal transplantation (Tang et al., 2017). 2) Liu et al. used a random forest model in comparison with other machine learning techniques or multiple linear regression to predict the pharmacogenetic-guided dosage of warfarin (Liu et al., 2015). 3) Li and colleagues evaluated the efficiency of random forest in comparison with multiple linear regression for the pharmacogenetic-guided dosage in Chinese patients (Li et al., 2015).		3) Yes
	To predict the occurrence/severity of adverse drug reactions.	1) Molassiotis et al. used a random forest model to cluster sign and symptoms that could predict the occurrence of nausea in patients receiving chemotherapy (Molassiotis et al., 2012). 2) Zhao et al. used a random forest to predict adverse drug event in electronic health records. The random forest provided a good performance that was increased by including historical data prior to the adverse drug event (Zhao et al., 2015). 3) Sudharsan et al. compared four different machine-learning techniques, including a random forest model, to predict hypoglycemia in patients with type 2 diabetes. The authors found that random forest was the best model to optimize for the prediction of the abovementioned event having a sensitivity of 92% and a specificity of 90% (Sudharsan et al., 2015). 4) Jeong et al. used a random forest model to predict adverse drug reactions in electronic healthcare records by using laboratory results as potential predictors (Jeong et al., 2018). 5) Hoang et al. used the random forest to identify drug safety signal in medication dispensing data (Hoang et al., 2018). 6) Larney and colleagues used a random forest model to identify patients at greater risk of adverse outcomes among those treated with opioid agonists (Larney et al., 2018).	2) Yes 6) Yes	3) Yes 4) Yes 5) Yes 6) Yes
	To predict drug-drug interactions	1) Hansen et al. applied a data-mining approach to identify warfarin-related drug-drug interactions in administrative registers. In particular, they used a random forest model to predict variable importance for the outcome. Authors were able to identify 7 out of 47 possible warfarin-drug interactions without a prior hypothesis (Hansen et al., 2016).		
	To predict drugs consumption	1) Devinsky et al. used a random forest model to predict treatment change (new, add-on or switch) in patients with epilepsy given a set of clinical variables (Devinsky et al., 2016). 2) Hu and colleagues found that random forest was the third best method in predicting drugs consumption for analgesia when compared to other machine learning techniques. The input variables in the model included a set of clinical and demographic features (Hu et al., 2012). 3) Shamir et al. used a random forest model to predict the correct treatment in patients with Parkinson exposed to deep brain stimulation (Shamir et al., 2015). 4) Simuni et al. used a random survival forest model to predict the time to initiation of symptomatic therapy patients with Parkinson disease (Simuni et al., 2016). Random survival forest is a variant of the abovementioned statistical technique that is used for right-censored data.	4) Yes	
	To predict the propensity score	1) Karim et al. found that random forest and other machine learning techniques such as hybrid methods such as Hybrid-LASSO or Hybrid-elasticNET perform better than standard pharmacoepidemiological methods (e.g. logistic regression) for confounder selection in the setting of high-dimensional propensity score (Karim et al., 2018). 2) Kern et al. used a random forest model to estimate the propensity score of receiving the combination budesonide/formoterol (Kern et al., 2015). 3) Wasko et al. used a random forest model to compute the propensity score or rather the probability of receiving prednisone rather than disease-modifying antirheumatic drugs (Chester Wasko et al., 2016). 4) Wasko et al. used a random forest model to compute the propensity score or rather the probability of receiving methotrexate rather than non-receiving methotrexate (Wasko et al., 2013).		
	To predict drug adherence and persistence	1) Hackshaw et al. used a random forest model to identify predictors of pazopanib persistence and adherence in patients that were naïve for this drug (Hackshaw et al., 2014).		
	To identify subpopulation more at risk of drug inefficacy	1) An et al. developed a random forest model to predict drug-resistant epilepsy using administrative claims data (An et al., 2018).		1) Yes
Bayesian additive regression tree	To predict the needed dosage given the patient’s characteristics	1) Tang et al. used a Bayesian an additive regression tree and other machine learning techniques to predict tacrolimus dose in patients underwent renal transplantation (Tang et al., 2017).		
	To predict adherence to pharmacological treatment	1) Lo-Ciganic et al. used Bayesian additive regression tree to predict medication adherence thresholds (Lo-Ciganic et al., 2015).	1) Yes	1) Yes
Bayesian machine learning	To predict the occurrence/severity of adverse drug reactions.	1) Lazic et al. used an ad-hoc Bayesian machine-learning model to predict hERG-mediated QT prolongation using information from drugs with known potential of increasing QT through hERG to train the model (Lazic et al., 2018).		
Bayesian network learning	To predict the clinical response following a pharmacological treatment	1) Cuypers et al. used a Bayesian network to identify interactions between drug-exposure, amino acid variants, and therapy response in patients with hepatitis C (Cuypers et al., 2017). 2) Schmitz et al. used a Bayesian network to identify genetic markers for treatment success in heart failure patients (Schmitz et al., 2014). Bayesian network learning provided a lower accuracy than other machine learning techniques used by the researchers. 3) Saadah et al. used a probabilistic network to identify the subpopulation of premature infants that benefit from the pharmacological prophylaxis with palivizumab. In particular, the authors found that the statistical method was able to identify two main features or rather extreme low-birth weight male infants and congenital heart disease as key elements for the effectiveness of the treatment (Saadah et al., 2014).		
	To predict adherence to pharmacological treatments	1) Anderson et al. used a Bayesian network to identify predictors of treatment adherence in patients with schizophrenia treated with atypical antipsychotics (Anderson et al., 2017).		
Convolutional neural network	To predict the occurrence/severity of adverse drug reactions.	1) Li et al. used the model to identify levodopa-induced dyskinesia in patients with Parkinson disease (Li et al., 2017).	1) Yes	
Decision table	To predict the clinical response following a pharmacological treatment	1) Schmitz et al. used a decision table to identify genetic markers for treatment success in heart failure patients (Schmitz et al., 2014). Decision table provided a lower accuracy than other machine learning techniques used by the researchers.		
Classification, regression and decision tree	To predict the clinical response following a pharmacological treatment	1) Pusch et al. used both classification and regression tree to identify clinical factors (e.g. therapy duration) associated with all-cause mortality in patients with extra-pulmonary tuberculosis (Pusch et al., 2014). 2) Sangeda et al. used a decision tree to predict the occurrence of virological failure in patients treated with antiretroviral drugs for HIV (Sangeda et al., 2014). 3) Yabu et al. used a decision tree to assess if immune and gene profiles can predict response to desensitization therapy in candidates for kidney transplantation (Yabu et al., 2016). 4) Go et al. used a decision tree to predict the response Vascular Endothelial Growth Factor Receptor (VEGFR)-Tyrosine Kinase Inhibitor (TKI) in patients with metastatic renal cell carcinoma (Go et al., 2019). 5) Podda et al. used a CART to predict platelet reactivity in clopidogrel-treated patients given a set of demographic and clinical information (Podda et al., 2017). 6) Banjar et al. used a CART to identify predictors of response to imatinib in patients with chronic myeloid leukemia (Banjar et al., 2017).	6) Yes	
	To predict the needed dosage given the patient’s characteristics	1) Tang et al. used a regression tree model together with other machine learning techniques to predict tacrolimus dose in patients undergoing renal transplantation (Tang et al., 2017). 2) Liu et al. used a regression tree model in comparison with other machine learning techniques or multiple linear regression to predict the pharmacogenetic-guided dosage of warfarin (Liu et al., 2015). 3) Li and colleagues evaluated the efficiency of classification and regression tree in comparison with multiple linear regression for the pharmacogenetic-guided dosage of warfarin discovering that for Chinese patients, the multiple linear regression gave the lowest mean absolute error (Li et al., 2015).		2) Yes 3) Yes
	To predict drug consumption	1) Hu et al. used a regression tree model machine to predict analgesic treatment (Hu et al., 2012).		1) Yes
	To predict the occurrence/severity of adverse drug reactions.	1) Hoang et al. used a regression tree model to identify drug safety signals in medication dispensing data (Hoang et al., 2018). 2) Sargent et al. used an xgboost algorithm to assess the association between anticholinergic drug burden and cognitive impairment, physical and cognitive frailty (Sargent et al., 2018).		1) Yes
	To predict adherence to pharmacological treatments	1) Franklin et al. used a boosted regression tree to predict treatment adherence (Franklin et al., 2016).		1) Yes
	To predict diagnosis leading to a drug prescription.	1) The decision tree has been used by Rezaei-Darzi et al. to predict the labeling diagnosis leading to a pharmaceutical prescription (Rezaei-Darzi et al., 2014).		1) Yes
K-means clustering	To predict the clinical response following a pharmacological treatment	1) Kan et al. used k-means cluster analysis to assess the association between longitudinal treatment patterns and the onset of clinical outcomes (Kan et al., 2016).		
K-nearest-neighbor	To predict the clinical response following a pharmacological treatment	1) deAndre´s-Galiana et al. used the k-nearest neighbors technique to identify prognostic variables for Hodgkin lymphoma treatment (deAndres-Galiana et al., 2015). 2) Albarakati and colleagues used a K-nearest-neighbor model to classify genes as interacting or not interacting with BRCA-1DNA repair gene among patients underwent to the pharmacological treatment with cisplatin for breast cancer (Albarakati et al., 2015). 3) Schmitz et al. used a K-nearest-neighbor model to identify genetic markers for treatment success in heart failure patients (Schmitz et al., 2014). The model provided the fourth best accuracy when compared to other machine learning techniques used by the researchers. 4) Podda et al. used this model to predict platelet reactivity in clopidogrel-treated patients given a set of demographic and clinical information.		
	To predict drug consumption	1) Hu et al. used the k-nearest-neighbor to predict analgesic treatment (Hu et al., 2012).		1) Yes
	To predict the occurrence/severity of adverse drug reactions.	1) Sudharsan et al. used a K-nearest-neighbor to predict hypoglycemia in patients with type 2 diabetes (Sudharsan et al., 2015).		1) Yes
Ridge, ElasticNET, and LASSO	To predict the clinical response following a pharmacological treatment	1) Tran et al. used penalized regression to estimate longitudinal treatment effects in simulated data and in a cohort of patients with HIV. Researchers found that weighted estimators performed better than covariate estimators did (Tran et al., 2019). 2) Yabu et al. used an elasticNET model to assess if immune and gene profiles can predict response to desensitization therapy in candidates for kidney transplantation (Yabu et al., 2016). 3) Ravanelli et al. used a LASSO regression to assess the predictive value of computed tomography texture analysis on survival in patients with lung adenocarcinoma treated with tyrosine kinase inhibitors (Ravanelli et al., 2018). 4) Saigo et al. used a LASSO regression to assess if the history of medical treatments predict anti-HIV therapy response (Saigo et al., 2011).		3) Yes 4) Yes
	To predict the needed dosage given the patient’s characteristics	1) Liu et al. used a LASSO regression in comparison with other machine learning techniques or multiple linear regression to predict the pharmacogenetic-guided dosage of warfarin (Liu et al., 2015).		
	To predict the propensity score	1) Karim et al. found that Hybrid-LASSO or Hybrid-elasticNET perform better than standard pharmacoepidemiological methods (e.g. logistic regression) for confounder selection in the setting of high-dimensional propensity score (Karim et al., 2018).		
	To predict the occurrence/severity of adverse drug reactions.	1) Larney and colleagues used the ridge/eleasticNET/LASSO regressions to identify patients at greater risk of adverse outcomes among those treated with opioid agonists (Larney et al., 2018).	1) Yes	1) Yes
Discriminant analysis	To predict the clinical response following a pharmacological treatment	1) Kohlmann et al. used both a linear and quadratic discriminant analysis to classify patients as resistant/non-resistant based on their longitudinal viral load profile (Kohlmann et al., 2009).		
Fuzzy-c-means	To predict the clinical response following a pharmacological treatment	1) Ravan et al. used the fuzzy-c-means algorithm to identify neurophysiologic changes induced by clozapine in patients with schizophrenia (Ravan et al., 2015).		
Naïve Bayes classifier	To predict the clinical response following a pharmacological treatment	1) Podda et al. used a Naïve Bayes classifier model to predict platelet reactivity in clopidogrel-treated patients given a set of demographic and clinical information (Podda et al., 2017). 2) Wolfson et al. used a naïve Bayes classifier to predict patients’ cardiovascular risk in the setting of time-to-event data both in simulated and real-world data (Wolfson et al., 2015).		
	To predict the occurrence/severity of adverse drug reactions.	1) Loke et al. used a naïve Bayes classifier model to predict the re-occurrence of severe chemotherapy-induced adverse drug reactions in patients with a medical history of this event (Loke et al., 2011). 2) Sudharsan et al. used a naïve Bayes classifier model to predict hypoglycemia in patients with type 2 diabetes (Sudharsan et al., 2015).		2) Yes
	To predict drugs consumption	1) Shamir et al. used a naïve Bayes classifier to predict the treatment in patients with Parkinson disease exposed to deep brain stimulation (Shamir et al., 2015). 2) Hu et al. used the k-nearest-neighbor to predict analgesic treatment (Hu et al., 2012).		2) Yes
Principal component analysis	To predict the clinical response following a pharmacological treatment	1) Yap et al. used the principal component technique to investigate anxiety characteristics that can predict the occurrence of chemotherapy-induced nausea and vomitting (Yap et al., 2012).		
Q-learning	To predict the clinical response following a pharmacological treatment	1) Krakow et al. used the Q-learning technique to identify the sequences of treatment regimens associated with improved survival (Krakow et al., 2017).		
	To optimize treatment regimen	1) Song et al. used the Q-learning technique to discover the optimal dynamic treatment regimen using data from a randomized trial for which the treatment regimens were randomized at multiple stages (Song et al., 2015).		
Support vector machine	To predict the clinical response following a pharmacological treatment	1) Ravan et al. used a support vector machine model to identify neurophysiologic changes induced by clozapine in patients with schizophrenia (Ravan et al., 2015). 2) Go et al. used a support vector machine model to predict the response VEGFR-TKI in in patients with metastatic renal cell carcinoma (Go et al., 2019). 3) Yabu et al. used a support vector machine model to assess if immune and gene profiles can predict response to desensitization therapy in candidates for kidney transplantation (Yabu et al., 2016). 4) Podda et al. used this model to predict platelet reactivity in clopidogrel-treated patients given a set of demographic and clinical information (Podda et al., 2017). 5) Albarakati et al. used a support vector machine model to predict genes that were expressed differently in patients with mRNA BRCA1+ and mRNA BRCA1− to assess their impact on prognosis (Albarakati et al., 2015). 6) Yun et al. used a support vector machine to assess if changes in cortical surface area or thickness predict the response to serotonin reuptake inhibitors in patients with obsessive-compulsive disorders (Yun et al., 2015). 7) Sun et al. used a support vector machine to assess the association between immunology biomarkers and the response to chemotherapy in patients with epithelial ovarian carcinoma (Sun et al., 2016). 8) Qin et al. used a support vector machine to examine the association between patterns of topological properties of brain network and major depressive disorders during their pharmacological treatment (Qin et al., 2015).	7) Yes 8) Yes	
	To predict the needed dosage given the patient’s characteristics	1) Tang et al. used a support vector machine together with other machine learning techniques to predict the tacrolimus dose in patients undergoing renal transplantation (Tang et al., 2017). 2) Guerrero et al. used a support vector machine to predict hemoglobin levels in order to adjust erythropoietin dosage among patients with chronic renal failure (Martin-Guerrero et al., 2003). 3) Li and colleagues evaluated the efficiency of a support vector machine in comparison with multiple linear regression for the pharmacogenetic-guided dosage of warfarin discovering in Chinese patients (Li et al., 2015).		3) Yes
	To predict drugs consumption	1) Shamir et al. used the support vector machine to predict the correct treatment in patients with Parkinson exposed to deep brain stimulation (Shamir et al., 2015). 2) Hu et al. used the support vector machine to predict analgesic treatment (Hu et al., 2012).	2) Yes	2) Yes
	To predict the occurrence/severity of adverse drug reactions.	1) Kesler et al. used the support vector machine to predict cognitive changes/deficits in patients with breast cancer that were/were not exposed to chemotherapy (Kesler et al., 2013). 2) Hoang et al. used the support vector machine to identify drug safety signal in medication dispensing data (Hoang et al., 2018). 3) Li et al. used the model to identify levodopa-induced dyskinesia in patients with Parkinson disease (Li et al., 2017). 4) Sudharsan et al. used a support vector machine to predict hypoglycemia in patients with type 2 diabetes (Sudharsan et al., 2015). 5) Jeong et al. used the support vector machine to predict adverse drug reactions in electronic healthcare records by using as potential predictors laboratory results (Jeong et al., 2018).		2) Yes 3) Yes 4) Yes 5) Yes
	To identify subpopulation more at risk of drug inefficacy	1) An et al. used the support vector machine to predict drug-resistant epilepsy using administrative claims data (An et al., 2018).		1) Yes
Kernel partial least squares	To predict the clinical response following a pharmacological treatment	1) Linke et al. used kernel partial least squares to investigate feature interaction while identifying predictors for clinical response in patients treated with tamoxifen for breast cancer (Linke et al, 2006; Yap et al., 2012).	1) Yes (specifically for features interaction)	
Hierarchical clustering	To predict the needed dosage given the patient’s characteristics	1) Berger et al. hierarchical clustering to identify predictors of the immune response to inﬂuenza vaccination (Berger et al., 2015).	1) Yes	

The main applications of artificial intelligence based knowledge discovery techniques in pharmacoepidemiology were classification/regression (44/72; 61.1%), classification/regression + model optimization (13/72; 18.0%), classification/regression + features selection (12/72; 16.7%), classification/regression + features interaction (1/72; 1.4%), and classification/regression + features selection + model optimization (2/72; 2.8%).

Classification and regression are two different types of predictive modeling where in the former the prediction is a label (class) whilst in the latter it is a quantity. For example, in classification, a patient can be classified as belonging to one of two classes: “having the disease” and “not having the disease” given a set of information from his/her medical history. In regression, instead, the researcher may try to predict the cholesterol level of a patient based on patient’s weight. Feature (variable) selection is a type of modeling in which the researcher constructs and trains statistical models by selecting relevant features to reduce overfitting and training time, and to improve accuracy. The main reason for feature selection is to improve the model performance that may be negatively impacted with the inclusion of partially relevant or irrelevant features as this leads to overfitting. Conversely, incorrectly excluding variables may lead to a bias in the model prediction (Heinze et al., 2018). Feature interaction, instead, is said to be relevant when the impact of any feature changes based on the levels of the other features hence rendering an additive model unsatisfactory. For a model with the lowest order interaction, the prediction is calculated based on a constant, a value for the first feature, a value for the second feature, and finally, the value for the interaction of the two features (Molnar, 2018).

In the retrieved articles, twenty different knowledge discovery techniques were used. Multiple techniques were used in the same article leading for a total of 122 applications. Random forest (30/122; 24.6%), artificial neural networks (22/122; 18.0%), and support vector machine (19/122; 15.6%) models were the three most used techniques (**Table 1**, **Supplementary Figure 2**). The top six purposes of using artificial intelligence techniques were to predict: 1) the clinical response following a pharmacological treatment (42.7%); 2) the occurrence/severity of adverse drug reactions (19.4%); 3) the needed dosage given the patient’s characteristics (14.5%); 4) drug consumption (9.7%), and 5) propensity score (4.8%) (**Table 1**).

### Lay Description of the Knowledge Discovery Techniques of Artificial Intelligence Used in Pharmacoepidemiology

#### Artificial Neural Network

An artificial neural network is a machine learning technique that tries to mimic neurons’ mechanisms of processing signals and is applicable to solve complex knowledge extraction tasks. In artificial neural networks, the input signals are characterized by the features variables (e.g., covariates) where each gets a different weight according to its importance in the knowledge extraction task (e.g., having or not having an adverse event). In its simplest form, as in the case of single-layer network, features represent the input nodes of the artificial neural networks, and all the input nodes are then arranged in one layer (e.g., skip-layer units) while the outcome represents the output node (Zhang, 2016a). Artificial neural networks can be split into two broad categories based on network topology, Feedforward and Feedback Artificial Neural Networks. The choice and applicability of the different network topology depend on the nature of problem. Convolutional Neural Network based on the principal of feedforward is well suited for the problems related to image analysis whereas problems such as speech recognition are better suited for the recurrent neural networks based on the feedback network topology. For this reason, the model has been used widely for computer vision task such as the automatic identification of patterns in medical images (Yamashita et al., 2018). Among studies selected in this systematic review, the artificial neural network was primarily used for Auto Contractive Maps (ACM). The ACM differs from the other artificial neural networks because it is able to learn from data without randomizing weight for each variable. In this technique, the weight of each variable is calculated based on their convergence criterion when all the output nodes become null. In particular, the model uses a data-driven mechanism to set-up weights based on the Euclidean space given the topological properties of each variable.

#### Bayesian Additive Regression Trees (BART)

BART is a technique that combines several Bayesian regression trees and starts by building an individual regression tree for each variable that are subsequently summed. By definition, the BART model is flexible and able to evaluate non-linear effects and multi-way interactions automatically. For each node of the regression tree, the levels of the variable are separated into two sub-groups based on their predictive power for the outcome. By definition, Bayesian additive regression trees are able to capture additive effects among variables (Hernandez et al., 2018).

#### Bayesian Network

A Bayesian network is a special machine learning technique used in causal inference. Causal inference determines the probability of an outcome using evidence from prior observations. The model use prior knowledge from a causal diagram (direct acyclic graph) which describes the underlying joint probability distribution among variables with conditional dependencies (Sesen et al., 2013). The model incorporates prior knowledge about the topic and then learns from the data how the variables interact with each other in the network.

#### Ridge, ElasticNET, and LASSO

In the case of high dimensional datasets where the number of variables is bigger than the number of observations, least squares method (linear model) cannot be used. In such a scenario, the commonly used approach is to reduce dimensionality through regularization. In such a case, penalized regression can be the preferred choice to perform feature selection. In this case the coefficients are obtained through the minimization of the penalized residual sum of squares where the penalty is imposed on the regression coefficients and used as a tuning parameter. If the penalty is imposed on the sum of the squared coefficents, penalized regression is called the Ridge regression. If the penalty is imposed on the sum of the absolute values of the coeeficients, we have the Least Absolute Shrinkage and Selection Operator (LASSO) regression. The Elastic Net imposes the penalty on the combination of the both sum of the squared and absolute values of the coefficients. LASSO forces (shrinks) the coefficients of all the variables with a poor contribution to the prediction to be zero and, therefore, these variables are excluded from the final model. ElasticNET, instead, shrinks some of the coefficient towards zero but also preserve some of the variables with medium-low predictive power providing a less aggressive feature selection strategy (Kyung et al., 2010).

#### Naïve Bayes Classifier

The naïve Bayes classifier is an artificial intelligence technique used for classification that relies on the Bayesian classification (Zhang, 2016c) based on the following principles: given the hypothesis h, a set of data D and a probability measure P, we can define P(h) as the probability that h is true. P(h) represents the prior knowledge on h; P(D) is the probability that the data in D will be observed; P(D|h) is the probability of observing the set D given that h is true; and P(h|D) is the probability that h true for a given data D, i.e., posterior probability of h. The theorem can be formalized as following: P(D|h) = P(D|h) P(h)/P(D). The theorem allows for calculating the posterior probability of h given D starting from the knowledge of the prior probabilities of D, and the conditional probability of D given h. Consequently, it is possible to calculate the maximum posterior hypothesis (MAP), or rather the most probable hypothesis of h given D. The naïve Bayes algorithm classifies the new data by assigning the most probable target value, or rather the MAP value, given the sequence of attributes (a_1_, a_2_,…, a_n_) that describe the new data.

#### Discriminant Analysis

A discriminant analysis is used to group observations based on the similarities of their features. Suppose we have *g* groups D_1_, D_2_,…, D*_g_* from which the observations are coming from. The objective of the discriminant analysis is to categorize an individual in one of these groups given a set of observations, *x*_1_, *x*_2, … … … … ,_ *x_p_* (where *p* is the number of variables). For example, we want to discriminate between patients with or without diabetes mellitus type 2 (g = 2) based on observations of glycaemia, body weight, and age (p = 3) (in this case x1 = blood glucose concentration, x2 = body weight, and *x*_3_ = age). For the specific characteristics of the individuals of a group *D_i_*, we can compute a probability that describes the likelihood of belonging to the group *i*, given the observed variables. Linear discriminant analysis is a classification technique that uses linear combinations of features to categorize observations in groups. The model requires that the data are normally distributed, homoscedastic or have an identical covariate matrix among classes. Quadratic discriminant analysis, instead, relaxes the last assumption or rather does not require that classes have the same covariate matrix.

#### Principal Component Analysis

The principal component analysis is a technique that reduces the dimensionality of quantitative variables in the dataset through linear combinations of these variables, also known as the principal components. The principal components are selected so that the first principal component (first linear combination) has the highest variance, the second principal component has the second highest variance but also uncorrelated with the first principal component and so on. When the original variables are highly correlated, only a few principal components are retained as they would still explain a large portion of the variation in the data.

#### Q-Learning

Q-learning is a reinforcement-learning algorithm used to optimize the solution of discrete time stochastic processes. The technique is “model-free” and “goal-oriented.” It provides at each stage of the process the optimal set of decisions to maximize a long-term reward. The algorithm is used in pharmacoepidemiology considering that many therapeutic processes are a set of actions that change over time and may be associated with a clinical outcome (i.e., a set of drugs administrated over time and the occurrence of an adverse drug reaction) (Song et al., 2015; Krakow et al., 2017).

#### Support Vector Machine and Sequential Minimal Optimization

Support vector machine (SVM) is a method used for classification. The SVM algorithm has three core components: i) A line; or a hyperplane as the “boundary” that separates data points, ii) A margin; i.e., the distance between the groups of data that are close to each other, and iii) Support vectors; i.e., the vectors to separate data points located within the margin of a hyperplane. In the presence of linearly separable data points, the algorithm finds among all straight lines or hyperplanes that separate the different groups those that maximize the margin value. In fact, a straight line or a hyperplane with maximum margin value allows minimizing the classification error. In non-linear classification, it is necessary to operate in two separate phases. In the first phase, data points are mapped on a large dimensional space to make them separable in a linear manner. Subsequently, the algorithm searches for a line or a hyperplane that maximizes the size of the margin, given that the instances are linearly separable. The support vector machine usually uses data transformations to transform a non-linear into a linear relationship of variables to simplify the delineation of boundaries. These data transformations usually use the kernel function (Noble, 2006). Sequential minimal optimization, instead, is an algorithm used to train the support vector machine (Platt, 1998).

#### Classification and Regression Tree

A classification and regression tree (CART) is a model constructed by recursively partitioning variables based on their predictive power for the study outcome. The model starts by identifying the variable with the strongest predictive power. This variable is included in the model as the root node or rather the parent node from which all other splitting procedures will be performed. In the regression tree, each node represents a variable. The decision tree split each node into two levels to make them have the best separation for maximizing their predictive power of the variable. With this model, the user does not need to make any assumptions about the statistical distribution of the data (e.g., normality assumption). The model can handle both categorical and numerical data (Kingsford and Salzberg, 2008). The boosted regression tree incorporates the important advantages of tree‐based method described above. However, it overcomes the inclusion of a single tree by including boosting (a combination of simple models to improve the overall predicting performance) (Elith et al., 2008).

#### Decision Table

A decision table is a hierarchical (rule) table used for classification in which attributes of variables are paired. A decision table is composed of columns with the inputs and outputs of a decision and rows denoting rules. This technique allows for the detection of the interrelationship among variables and their attributes (Becker, 1998). Decision tables use the wrapper method that finds the best subset of features or rather it removes features with a poor contribution to the model. In this way, the algorithm reduces the probability of overfitting.

#### K-Means Cluster

The k-means clustering algorithm uses unlabeled data to generate a fixed number (k) of clusters of data with similarities in attributes. The center of the clusters (k) is called centroids and are calculated by averaging data allocated to the cluster. The algorithm is composed of two steps: 1) Initialization, where the user sets the number of clusters, k, 2) the application of an algorithm (e.g. Lloyd’s algorithm) for which each data point is assigned to its closest cluster (Bock, 2007). The process iterates until the variation of data points in the cluster is minimized.

#### K-Nearest Neighbors

K-nearest neighbors is a machine learning technique used for both regression and classification. The k-Nearest Neighbor algorithm uses a training dataset with labeled data to classify new data points without labels. In the training dataset, the number of clusters (k) is identified based on their labels (e.g., having or not having a disease). The algorithm classifies a new data point by calculating its distance to each cluster of the training set until the closest cluster is identified. The technique does not make any assumption about the distribution of data (Zhang, 2016b).

#### Fuzzy C-Means

The fuzzy c-means is an artificial intelligence technique for clustering based on the similarities in the features. The term fuzzy stands for indistinct, confused, and blurred. It is based on the assumption that the world around us is not dichotomous (e.g., black and white) but contains in itself all the infinite nuances that exist between these two extremes. This concept is expressed mathematically by a real number between zero and one that represents the degree of membership (membership function) of the object in question to one or the other group (e.g., how much a gray is white, or how much a gray is black).

#### Random Forest and Random Survival Forest

Random forest is a machine leaning method based on the principle of ensemble learning. The key aspiration behind the random forest is to improve the performance of the indvidual tree learners with the help of bootstrap aggregating (or bagging). The technique builds each tree by bootstrapping a random sample from the data. To select the variables that need to be split in the decision tree, the random forest randomly selects features and uses scores (e.g., the decrease in Gini impurity score) as the splitting criterion. Gini impurity is a metric used in decision trees to determine which variable and at what threshold the data should be split into smaller groups. Gini Impurity measures misclassification of random records from the data set used to train the model. To understand the importance of each variable for classification/regression, the random forest classifies variables based on their importance for classification/regression in a parameter called “variable importance measure,” which has however been noted to be biased. Alternative measures are available to overcome this limitation, such as partial dependent plots. These plots provide an overview of how each variable influences the prediction of the study outcome when related to other variables selected by the random forest. Crucial parameters for the random forest are the number of trees generated in the random forest, the number of variables randomly selected for splitting in each decision tree, and the minimum size of each terminal node (Couronne et al., 2018).

#### Kernel Partial Least Squares

Kernel partial least squares is a nonlinear partial least squares (PLS) method. PLS is a dimensionality reduction technique that models independent variables using latent variables (also known as components as in PCA). The aim is to find a few linear combinations of the original variables that are most correlated with the output. This technique is able to minimize multicollinearity among variables and it is useful in the set of high-dimensional datasets (Rosipal and Trejo, 2001).

#### Hierarchical Clustering

Hierarchical clustering is a technique that performs a hierarchal decomposition of the data based on group similarities. The model builds up a distance matrix that computes the distance among data points. In particular, given a set of N observations to be grouped, and a distance (or similarity) matrix N × N, which defines the distance of the data points to each other, the basic process of hierarchical grouping is as follows:

1. The algorithm starts associating a cluster to each entity so it will have initially N clusters, each of which contains only one data point and then computes the distance (similarity) among the clusters.
2. Subsequently, it will look for the pair of clusters that are “close” to each other (more similar) and it will combine them in a single cluster. In this way, the number of clusters will be reduced by one unit.
3. It will calculate again the distance (similarity) between the new cluster and each of the old clusters.
4. It will repeat steps 2 and 3 until the entities are grouped in the desired cluster number (Johnson, 1967).

## Discussion

In the last decade, there has been increased use knowledge discovery techniques of artificial intelligence in pharmacoepidemiology. This result is in line with those of Koohy (2017) who showed an increased popularity of machine learning methods for biomedical research from 1990 to 2017. We strongly believe that one of the major consequences for the increased interest in applying machine learning techniques over the years is the dramatic growth in size and complexity of clinical and biological data that have led to the necessity of combining mathematics, statistics, and computer science to extract actionable insight. By using advanced algorithms that are capable of self-learning from the data, machine-learning techniques provide support for decision making to the final user (e.g. a researcher) without a pre-specific hypothesis (i.e., “hypothesis-free algorithms”). In this systematic review, we found that random forest, artificial neural network, and support vector machine were the most used techniques in the selected articles. The extensive use of artificial neural networks may be related to its first appearance in the scientific literature. In fact, this technique has existed for over 60 years (Jones et al., 2018). Random Forest instead, since its introduction in 2001 (Breiman, 2001), has rapidly gained popularity becoming a common “standard tool” to predict clinical outcomes with the advantage of being easily usable by scientists without any strong knowledge in statistics or machine learning (Couronne et al., 2018). Similarly, the support vector machine is considered to be one of the most powerful techniques for the recognition of subtle patterns in complex datasets (Huang et al., 2018). Interestingly, we observed that in the majority of the articles, researchers used more than one knowledge discover technique, which is a common approach in large data analytics. In fact, it is usually not possible to know beforehand the best algorithm for a specific classification/regression progress, and data scientist should rely on “past experience from other scientists” or benchmark multiple algorithms in order to determine the one that maximizes the accuracy of the model, an approach also known as “use trial and error” (Brownlee, 2014).

It should be highlighted that we found that secondary data were mostly used among selected articles. This is not surprising considering that electronic healthcare databases and administrative databases have revolutionized pharmacoepidemiology research in the last three decades. These data sources can be used by pharmacoepidemiologists to address clinical questions on drug use, drug effectiveness, and treatment optimization (Hennessy, 2006) carrying the advantage of being easier and less costly to reuse than primary data that, on the contrary, required to be collected anew (Schneeweiss and Avorn, 2005).

As expected, the majority of selected articles provided a medium-low level of evidence according to the Merlin scale (Merlin et al., 2009), a phenomenon that is a natural consequence of the level of evidence that is attributed to observational studies (Murad et al., 2016). In fact, among selected articles, the majority used a cohort or a case-control design, therefore, independently of the technique that was used to predict the study outcome the level of evidence was classified as medium-low.

In the selected articles, we identified 17 medical fields, of which the most prevalent were pure pharmacoepidemiology (mostly methodological studies in pharmacoepidemiology), oncology, infective medicine, and neurology. Clearly, the high frequency of articles investigating pure pharmacoepidemiology is related to the research query used for selecting the articles. Regarding the other medical fields, our findings are in accordance with the current scientific literature (Jiang et al., 2017). In fact, a recent article showed increased use of artificial intelligence in areas with a high prevalence of the disease of which an early diagnosis may guarantee a better prognosis or a reduced disease progression like oncology, neurology, and cardiology.

Finally, it is not surprising that the main purpose of using artificial intelligence techniques in this systematic review was related to the prediction of a clinical response to a treatment (i.e., supervised learning problems). Artificial intelligence and machine learning techniques have entailed some important methodological advancements in the analysis of “big data.” The utility of these techniques lies behind their potential for analysing large and complex data for making predictions that can improve and personalize the management and treatment of a disease, and improve the total well-being of an individual (Collins and Moons, 2019). As secondary purpose of using artificial intelligence techniques there was the prediction of occurrence/severity of adverse drug reactions. In this case, it can be related to the great impact of adverse drug reactions as iatrogenic disease that requires often a treatment and represents a cost to the health-care system.

## Conclusion

The use of knowledge discovery techniques from artificial intelligence has increased exponentially over the years covering numerous sub-topics of pharmacoepidemiology. Random forest, artificial neural networks, and support vector machine models were the three most used techniques applied mainly on secondary data. The aforementioned techniques have been used mostly to predict the clinical response following a pharmacological treatment, the occurrence/severity of adverse drug reactions and the needed dosage is given the patient’s characteristics.

In the second part of this systematic review, we will summarize the evidence on the performance of artificial intelligence versus traditional pharmacoepidemiological techniques.

## Author Contributions

All authors drafted the paper, revised it for important intellectual content, and approved the final version of the manuscript to be published. MS and MA developed the concept and designed the study. MS, DL, MA, MK, and AK analyzed or interpreted the data. MS, DL, MA, MK, and AK wrote the paper.

## Funding

Maurizio Sessa, David Liang, and Morten Andersen belong to the Pharmacovigilance Research Center, Department of Drug Design and Pharmacology, University of Copenhagen, supported by a grant from the Novo Nordisk Foundation (NNF15SA0018404).

## Conflict of Interest

The authors declare that the research was conducted in the absence of any commercial or financial relationships that could be construed as a potential conflict of interest.
