# Supplementary figures and images for: Artificial Intelligence in Pharmacoepidemiology: A Systematic Review. Part 1—Overview of Knowledge Discovery Techniques in Artificial Intelligence
Source: Front Pharmacol. 2020 Jul 16;11:1028. doi: 10.3389/fphar.2020.01028 (PMC7378532; doi:10.3389/fphar.2020.01028)

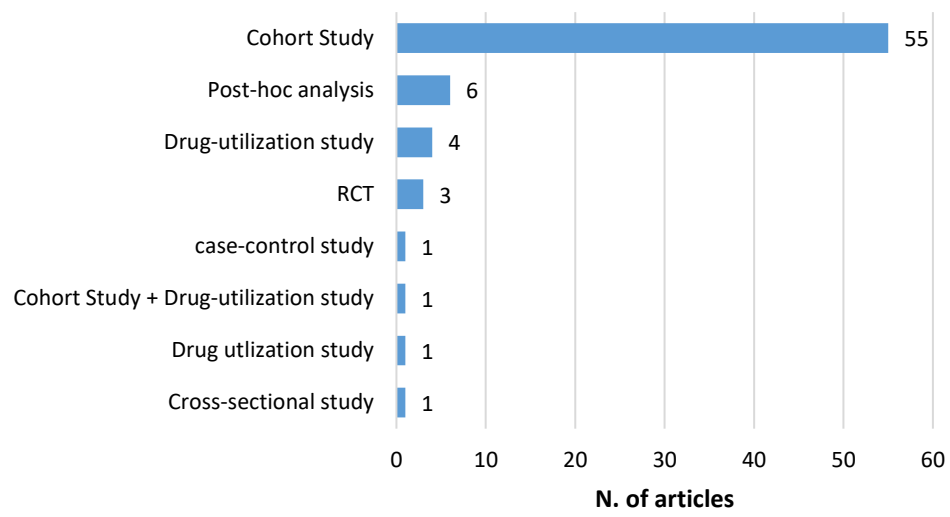

**Supplementary Figure 1.** Artificial intelligence techniques stratified by study design.

Supplement: Supplementary file 1 [file Image_1.pdf]

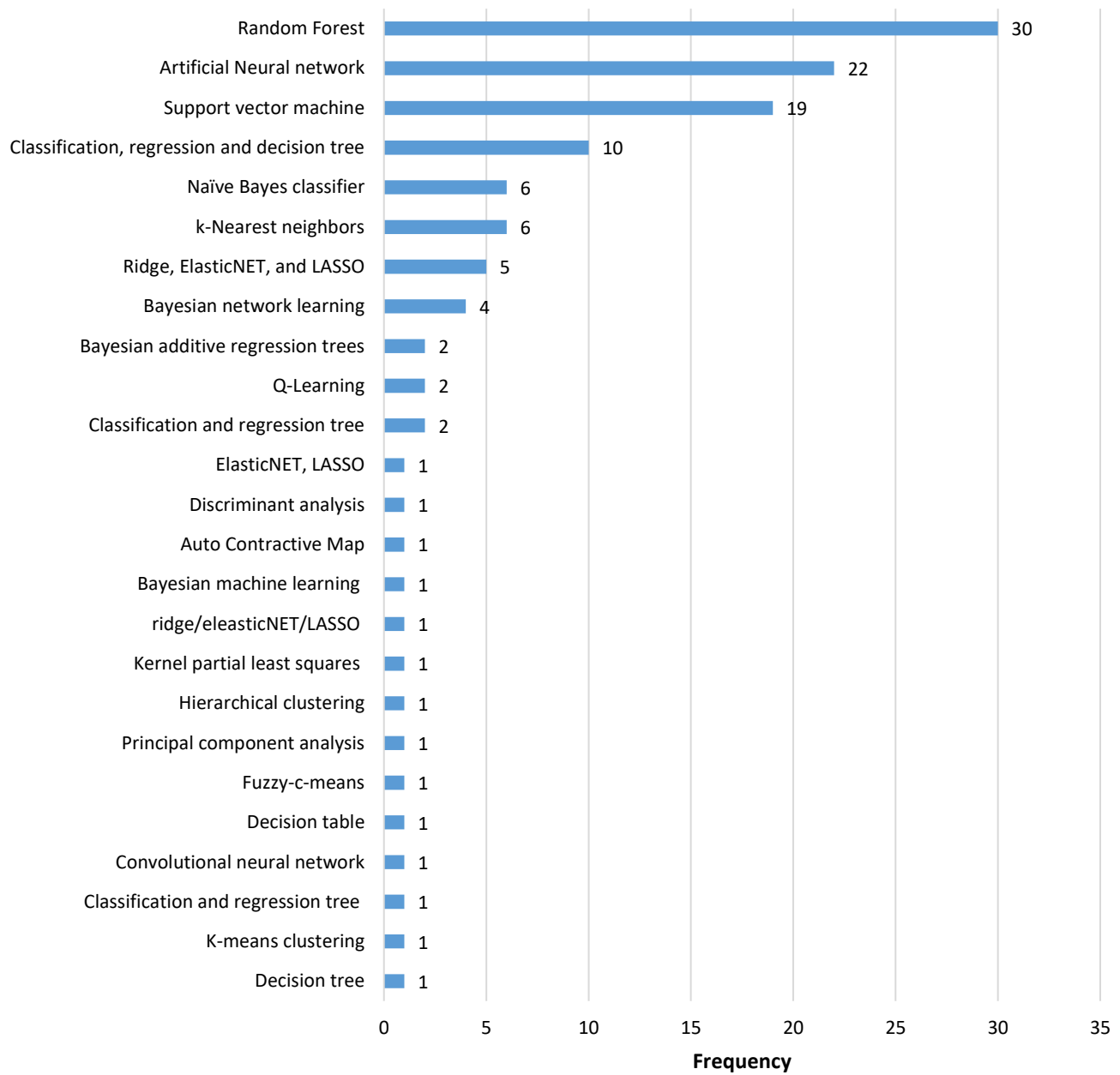

**Supplementary Figure 2.** Artificial intelligence techniques used.

Supplement: Supplementary file 2 [file Image_2.pdf]
