# Supplementary material for: Artificial Intelligence in Pharmacoepidemiology: A Systematic Review. Part 1—Overview of Knowledge Discovery Techniques in Artificial Intelligence
Source: Front Pharmacol. 2020 Jul 16;11:1028. doi: 10.3389/fphar.2020.01028 (PMC7378532; doi:10.3389/fphar.2020.01028)
Supplement: Supplementary file 3 [file Table_1.docx]

**Supplementary Table 1**. Research query.

| **PubMed Block Research Query – performed on 08/05/2019 at 09.32 GMT +2** | | | |
| --- | --- | --- | --- |
| **Blocks** | **Query** | **N. of articles** | **Reference** |
| Final research block | ((("1950/01/01"[Date - Publication] : "2019/05/06"[Date - Publication])) AND (((((((((((((((((((deep learning[Title/Abstract]) OR representation learning[Title/Abstract]) OR neural network*[Title/Abstract]) OR convolutional neural network*[Title/Abstract]) OR ConvNet[Title/Abstract]) OR CNN[Title/Abstract]) OR recurrent neural network*[Title/Abstract]) OR RNN[Title/Abstract]) OR long short-term memory[Title/Abstract]) OR LSTM[Title/Abstract]) OR generative adversarial network*[Title/Abstract]) OR GAN[Title/Abstract]) OR autoencoder[Title/Abstract]) OR restricted boltzmann machine*[Title/Abstract]) OR deep belief network*[Title/Abstract]) OR DBN[Title/Abstract])) OR ((((((“Artificial Intelligence”) OR (“computer analysis” OR “computer evaluation”)))) OR ((“Big Data” [MeSH] OR “Data Science” [MeSH] AND “Intelligence, Artificial” [MeSH] OR “Computational Intelligence” [MeSH] OR “Intelligence, Computational” [MeSH] OR “Machine Intelligence” [MeSH] OR “Intelligence, Machine” [MeSH] OR “Computer Reasoning” [MeSH] OR “Reasoning, Computer” [MeSH] OR “AI (Artificial Intelligence)” [MeSH] OR “Computer Vision Systems” [MeSH] OR “Computer Vision System” [MeSH] OR “System, Computer Vision” [MeSH] OR “Systems, Computer Vision” [MeSH] OR “Vision System, Computer” [MeSH] OR “Vision Systems, Computer” [MeSH] OR “Machine Learning” [MeSH].))) OR ((((((((((((((Artificial intelligence[Title/Abstract]) OR Bayesian learning[Title/Abstract]) OR Boosting[Title/Abstract]) OR Computational intelligence[Title/Abstract]) OR Computer reasoning[Title/Abstract]) OR Deep learning[Title/Abstract]) OR Machine intelligence[Title/Abstract]) OR Machine learning[Title/Abstract]) OR Naive Bayes[Title/Abstract]) OR Neural network[Title/Abstract]) OR Neural networks[Title/Abstract]) OR Natural language processing[Title/Abstract]) OR Support vector*[Title/Abstract]) OR Random forest*[Title/Abstract]))))) AND ((((((((((((((epidemiologic studies/) OR exp case-control studies/) OR exp cohort studies/) OR cross-sectional studies/) OR ((epidemiologic adj (study OR studies)) .ab, ti.)) OR case control.ab, ti.) OR cross sectional) OR cohort analg) OR ((follow up (study OR studies)) .ab, ti.)) OR longitudinal) OR ((observ$ adj3 (study OR studies)) .ab, ti.)) OR adverse effect?.ab, ti.)) OR ((pharmacovigilance OR Adverse Drug Reaction Reporting Systems[MH] OR pharmacovigilan*[ti] OR Drug-Related Side Effects and Adverse Reactions[MH] OR adverse[ti] OR toxicit*[ti]))) | 6470  08/05/2019 at 09.32 GMT +2 |  |
| Block 4 - period | ("1950/01/01"[Date - Publication] : "2019/05/06"[Date - Publication]) | 29099197  08/05/2019 at 09.32 GMT +2 |  |
| Block 3 – Artificial intelligence, machine learning or deep learning | (((((((((((((((((deep learning[Title/Abstract]) OR representation learning[Title/Abstract]) OR neural network*[Title/Abstract]) OR convolutional neural network*[Title/Abstract]) OR ConvNet[Title/Abstract]) OR CNN[Title/Abstract]) OR recurrent neural network*[Title/Abstract]) OR RNN[Title/Abstract]) OR long short-term memory[Title/Abstract]) OR LSTM[Title/Abstract]) OR generative adversarial network*[Title/Abstract]) OR GAN[Title/Abstract]) OR autoencoder[Title/Abstract]) OR restricted boltzmann machine*[Title/Abstract]) OR deep belief network*[Title/Abstract]) OR DBN[Title/Abstract])) OR ((((((“Artificial Intelligence”) OR (“computer analysis” OR “computer evaluation”)))) OR ((“Big Data” [MeSH] OR “Data Science” [MeSH] AND “Intelligence, Artificial” [MeSH] OR “Computational Intelligence” [MeSH] OR “Intelligence, Computational” [MeSH] OR “Machine Intelligence” [MeSH] OR “Intelligence, Machine” [MeSH] OR “Computer Reasoning” [MeSH] OR “Reasoning, Computer” [MeSH] OR “AI (Artificial Intelligence)” [MeSH] OR “Computer Vision Systems” [MeSH] OR “Computer Vision System” [MeSH] OR “System, Computer Vision” [MeSH] OR “Systems, Computer Vision” [MeSH] OR “Vision System, Computer” [MeSH] OR “Vision Systems, Computer” [MeSH] OR “Machine Learning” [MeSH].))) OR ((((((((((((((Artificial intelligence[Title/Abstract]) OR Bayesian learning[Title/Abstract]) OR Boosting[Title/Abstract]) OR Computational intelligence[Title/Abstract]) OR Computer reasoning[Title/Abstract]) OR Deep learning[Title/Abstract]) OR Machine intelligence[Title/Abstract]) OR Machine learning[Title/Abstract]) OR Naive Bayes[Title/Abstract]) OR Neural network[Title/Abstract]) OR Neural networks[Title/Abstract]) OR Natural language processing[Title/Abstract]) OR Support vector*[Title/Abstract]) OR Random forest*[Title/Abstract])) | 113579  08/05/2019 at 09.32 GMT +2 | (Balayla and Shrem, 2019; Mak et al., 2019; Yannick et al., 2019) |
| Block 2 - Pharmacovigilance | (pharmacovigilance OR Adverse Drug Reaction Reporting Systems[MH] OR pharmacovigilan*[ti] OR Drug-Related Side Effects and Adverse Reactions[MH] OR adverse[ti] OR toxicit*[ti]) | 228612  08/05/2019 at 09.32 GMT +2 | (Baldo et al., 2018) |
| Block 1 - epidemiology | (((((((((((epidemiologic studies/) OR exp case-control studies/) OR exp cohort studies/) OR cross-sectional studies/) OR ((epidemiologic adj (study OR studies)) .ab, ti.)) OR case control.ab, ti.) OR cross sectional) OR cohort analg) OR ((follow up (study OR studies)) .ab, ti.)) OR longitudinal) OR ((observ$ adj3 (study OR studies)) .ab, ti.)) OR adverse effect?.ab, ti. | 2560636  08/05/2019 at 09.32 GMT +2 | (Li et al., 2019) |

Unless otherwise stated, search terms are free text terms; MeSH = Medical subject heading (Medline medical index term); exp = exploded MeSH; the dollar sign ($) stands for any character(s); the question mark (?) = to substitute for one or no characters; tw = text word; pt = publication type; sh = MeSH; adj = adjacent.
