# Supplementary material for: Artificial Intelligence in Pharmacoepidemiology: A Systematic Review. Part 1—Overview of Knowledge Discovery Techniques in Artificial Intelligence
Source: Front Pharmacol. 2020 Jul 16;11:1028. doi: 10.3389/fphar.2020.01028 (PMC7378532; doi:10.3389/fphar.2020.01028)
Supplement: Supplementary file 6 [file Table_4.docx]

**Supplementary table 4**. Medical fields.

| **Medical field** | **N. of articles** | **%** |
| --- | --- | --- |
| Diabetology | 1 | 1.4% |
| Geriatrics | 1 | 1.4% |
| Dermatology | 1 | 1.4% |
| Anesthesiology | 1 | 1.4% |
| Psychiatric | 1 | 1.4% |
| Pneumology | 1 | 1.4% |
| Gastroenterology | 2 | 2.8% |
| Reumathology | 2 | 2.8% |
| Nephrology | 3 | 4.2% |
| Cardiology | 3 | 4.2% |
| Psychiatry | 3 | 4.2% |
| Immunology | 4 | 5.6% |
| Pharmacogenetics | 4 | 5.6% |
| Neurology | 6 | 8.3% |
| Infective medicine | 8 | 11.1% |
| Oncology | 15 | 20.8% |
| Pure pharmacoepidemiology | 16 | 22.2% |
