# Supplementary material for: Artificial Intelligence in Pharmacoepidemiology: A Systematic Review. Part 1—Overview of Knowledge Discovery Techniques in Artificial Intelligence
Source: Front Pharmacol. 2020 Jul 16;11:1028. doi: 10.3389/fphar.2020.01028 (PMC7378532; doi:10.3389/fphar.2020.01028)
Supplement: Supplementary file 7 [file Table_5.docx]

**Supplementary Table 5**. Specific data sources.

| **Data sources** | **N. of articles** |
| --- | --- |
| (DOI: 10.1067/mcp.2002.121775) + (https://doi.org/10.1067/mhj.2002.119769) + (J Pharmacol Exp Ther. 2001 May;297(2):496-500.) | 1 |
| 10 North American rheumatology practices (Arthritis, Rheumatism, and Aging Medical Information System study) | 1 |
| 10.1016/j.jpainsymman.2011.11.003 | 1 |
| 10.1109/TBME.2003.816084 | 1 |
| 3 Italian centers | 1 |
| Acute HIV Infection and Early Disease Research Program (AIEDRP) | 1 |
| Arthritis, Rheumatism, and Aging Medical Information Systems study and US National Death Index | 1 |
| Asan Medical Center | 1 |
| Australian Pharmaceutical Benefit Scheme (PBS) | 1 |
| Center for International Blood and Marrow Transplant Research | 1 |
| Changhwa Christian Hospital (CCH). | 1 |
| Cleveland university hospital | 1 |
| Clin Transl Oncol (2015) 17:612–619 (DOI 10.1007/s12094-015-1285-z) | 1 |
| Clinical Practice Research Datalink and Hospital Episode Statistics database | 1 |
| Community behavioral health organizations | 1 |
| CVS Caremark, a U.S. pharmacy benefits management company | 1 |
| Danish administrative healthcare registries. | 1 |
| Diabetes Care. 2011;34(9):1934-1942 | 1 |
| doi.org/10.1002/mds.10310 | 1 |
| doi.org/10.1016/j.ejrad.2018.10.016 | 1 |
| doi.org/10.1093/toxsci/kfx125 | 1 |
| doi: 10.1016/j.clgc.2011.10.001 | 1 |
| DOI: 10.1038/srep42192 | 1 |
| DOI: 10.1089/10915360152745858 | 1 |
| DOI: 10.1097/JCP.0b013e31824888a1 | 1 |
| doi: 10.1097/MEG.0b013e3283424e3e | 1 |
| DOI: 10.1097/PRS.0000000000005257 | 1 |
| doi: 10.1109/EMBC.2017.8037580. | 1 |
| DOI: 10.1691/ph.2012.2633 | 1 |
| dx.doi.org/10.1016/j.clinph.2014.07.017 | 1 |
| Electronic Reporting and Recording of Controlled Drugs (ERRCD) system | 1 |
| electronic State of Texas surveillance database | 1 |
| EuResist integrated database | 1 |
| Fresenius Medical Care (FME) clinics in Portugal, Spain and Italy | 1 |
| GEPRESS | 1 |
| HealthCore Integrated Research Environment (HIRE) | 1 |
| HIV/AIDS Care and Treatment Centre (CTC) that provides ART at Amana District Hospital in Dar es Salaam, Tanzania AND Swiss HIV Cohort Study Adherence Questionnaire (SHCS-AQ) | 1 |
| HMO Research Network Virtual Data Warehouse (HMORN VDW) | 1 |
| IMS Health Surveillance Data Incorporated (SDI) medical claims database | 1 |
| InCHIANTI baseline 90-minute interview | 1 |
| IQVIA | 1 |
| Iranian health insurance organizations (SSIO, MSIO, and AFMSIO) | 1 |
| Italy (Department of Experimental Medicine and Surgery of the University of Tor Vergata, Rome), Slovenia (University of Ljubljana) and Belgium(University Hospitals Leuven, Leuven) | 1 |
| MarketScan commercial claims database (Truven Health Analytics, Ann Arbor, Michigan) | 1 |
| Nanjing Brain Hospital | 1 |
| National Cancer Centre Singapore (NCCS) | 1 |
| Neurobiol Dis 48(3): 329–338. | 1 |
| Nottingham Tenovus Primary Breast Carcinoma series | 1 |
| Observational Health Data Science and Informatics (OHDSI), EU-SPC and SIDER databases | 1 |
| OCD clinic at Seoul National University Hospital (SNUH) | 1 |
| Parkinson’s Progression Markers Initiative (PPMI) database | 1 |
| Pennsylvania Medicaid administrative claims data | 1 |
| Pharmacogenetics and Pharmacogenomics Knowledge Base | 1 |
| Pharmacogenetics and Pharmacogenomics Knowledge Base (PharmGKB, www. pharmgkb.org). Speciﬁcally, The International Warfarin Pharmacogenetics Consortium | 2 |
| Simulated data | 1 |
| SMART | 1 |
| Stockholm EPR Corpus (Karolinska University Hospital) | 1 |
| Study ID SDY788 (DOI:10.1371/journal.pone.0153355) | 1 |
| Sun Yat-Sen Memorial Hospital | 1 |
| tertiary care hospital in the United Arab Emirates | 1 |
| TIDAL II Clinical Trial | 1 |
| Tongji Hospital and Hubei Cancer Hospital | 1 |
| Truven Health MarketScan Commercial Claims and Encounters Database AND Truven Health MarketScan Medicare Supplemental and Coordination of Benefits Database | 1 |
| Türkiye Yüksek İhtisas Hospital Inflammatory Bowel Disease clinic | 1 |
| United Kingdom data from the Clinical Practice Research Datalink and Hospital Episode Statistics database | 1 |
| University Hospital Basel, Switzerland | 1 |
| University Hospital in Basel (Switzerland), the Women’s Hospital Rheinfelden (Germany), and the Kreiskrankenhaus Lo¨rrach (Germany) | 1 |
| University of Gondar Hospital | 1 |
| University of Louisville ( doi.org/10.1046/j.1525-1594.1998.06101.x ) | 1 |
| University of Michigan Health System Data Warehouse | 1 |
| Yonsei University College of Medicine, Seaul, Korea | 1 |
| **Total** | **72** |
